# Effects of Prolactin on Brain Neurons under Hypoxia

**DOI:** 10.3390/life14010152

**Published:** 2024-01-21

**Authors:** Naoto Tani, Tomoya Ikeda, Takaki Ishikawa

**Affiliations:** 1 Department of Legal Medicine, Graduate School of Medicine, Osaka Metropolitan University, 1-4-3 Asahi-machi, Abeno, Osaka 545-8585, Japan; tikeda@cc.saga-u.ac.jp (T.I.); takaki@omu.ac.jp (T.I.); 2 Forensic Autopsy Section, Medico-Legal Consultation and Postmortem Investigation Support Center, 1-4-3 Asahi-machi, Abeno, Osaka 545-8585, Japan

**Keywords:** prolactin, brain, neuroprotection, hypoxia, forensics

## Abstract

The levels and potential role of prolactin (PRL) in the brain under conditions of acute systemic hypoxia were examined, focusing on the accumulation of PRL in cerebrospinal fluid (CSF) and its effects on neuronal activity and injury. The amount of PRL in the brain was investigated using brain tissues from forensic autopsy cases. We counted the number of neurites that formed in human primary neurons (HNs) after the addition of PRL. Furthermore, HNs supplemented with PRL or triiodothyronine (T3) were exposed to hypoxic conditions, and the dead cells were counted. The results showed correlations between brain PRL and CSF PRL levels. Additionally, PRL accumulation in the brain was observed in cases of asphyxia. In vitro experimental findings indicated increased neurite formation in the HNs treated with PRL. Moreover, both PRL and T3 demonstrated neuroprotective effects against hypoxia-induced neuronal cell death, with PRL showing stronger neuroprotective potential than T3. These results suggest that PRL accumulates in the brain during hypoxia, potentially influences neuronal activity, and exhibits neuroprotective properties against hypoxia-induced neuronal injury.

## 1. Introduction

Prolactin (PRL), except for placental PRL, is a hormone that is mainly synthesized and secreted by lactotroph cells in the anterior pituitary gland [1]. PRL accumulates in the tissue microenvironment and elicits its actions in an autocrine or paracrine manner to regulate diverse physiological activities, including the immune response, osmotic pressure, angiogenesis, and promotion of neurogenesis in the maternal and fetal brains [2,3,4,5,6]. PRL is transported to the brain via specific transporters expressed in the choroid plexus [7], but it has also been reported to be produced locally in the brain [8,9,10,11]. The PRL receptor (PRLR) belongs to the type I cytokine receptor family and consists of three domains: extracellular, transmembrane, and intracellular [12,13]. In addition, expression of PRLR has been reported in various brain regions, including the cerebral cortex, hypothalamus, and hippocampus in mammals [13]. Regarding the binding of PRL to the single-pass, the transmembrane PRLR induces several intracellular signaling cascades that are mediated by the Janus kinase (JAK)-signal transducer and activator of transcription (STAT) components [14]. Specifically, JAK2 is constitutively associated with PRLR, and once JAK2 is activated, it recruits and phosphorylates STAT5 [15]. STAT5 regulates the expression of several target genes in the nucleus, including genes related to cell cycle and survival [16].

Several studies on hyperprolactinemia under conditions of hypoxemia have been reported [17,18,19]. In addition, other pathological conditions that have been reported to be associated with elevated PRL levels include physiological and physical stress (trauma), such as burns, surgery, and post-traumatic stress disorder [20]. In our previous study, we reported that PRL levels were increased in the cerebrospinal fluid (CSF) during acute systemic hypoxia due to asphyxia, and PRL was transported from the blood to the CSF via the choroid plexus under hypoxia [21]. However, the physiological significance of the PRL transported to the CSF under hypoxia remains unclear.

As a result of hypoxia/ischemia in the brain, brain edemas and inflammation occur, leading to hypoxic-ischemic encephalopathy (HIE) [22]. In forensic autopsy cases, HIE can be caused by multiple conditions, such as traumatic or chemical events, respiratory and cardiac arrest, asphyxiation, or obstruction of the cerebral or cervical vessels [23,24]. In addition, neonatal HIE is one of the major causes of neurodegeneration and death in the neonatal period [25]. Treatment is primarily by hypothermia, but the use of neuroprotective peptides targeting cytotoxic or cytoprotective pathways has been reported [26]. For example, it has been reported that triiodothyronine (T3) may be involved in the neuroprotective mechanism of hypoxic preconditioning that resulted in potent neuroprotection against HIE [27]. It has also been reported that PRL has complex stimulatory and regulatory effects on neural stem cell activity and may itself play a role in the recovery process associated with damage in the brain via signal transduction through PRLR [28]. PRL is also expected to be a neuroprotective agent, but its role in humans during hypoxia is not fully understood, and in vivo studies are lacking [29].

In the present study, we examined the levels and potential role of PRL in the brain under conditions of acute systemic hypoxia with a focus on PRL accumulation in the CSF and its effects on neuronal activity and injury.

## 2. Materials and Methods

### 2.1. Ethics Statement

The protocol of the present study was evaluated and approved by the Independent Ethics Committee of the Osaka Metropolitan University Graduate School of Medicine. An opt-out form of informed consent was approved for the use of autopsy data for analysis (Authorization no. 4087).

### 2.2. Autopsy Samples

Serial autopsy cases were examined within 72 h postmortem at our institution. There were 104 cases, and the median age was 67 years (range, 0–100 years). Cases were excluded from this study if any drugs were detected. The specimens were collected aseptically using syringes. Blood was collected from the right heart chamber. The blood samples were immediately centrifuged to separate the serum and then stored at −20 °C until use. The cause of death was classified according to the findings of the complete autopsy and macromorphological, micropathological, and toxicological examinations as follows: sharp instrument injury (*n* = 12), blunt injury (*n* = 32), fire fatality (*n* = 26), asphyxia (*n* = 20), drowning (*n* = 6), and acute cardiac death (*n* = 8). The case profiles are presented in Table 1. For each cause of death, clear and verifiable cases with well-established pathological evidence without any significant complications were included.

### 2.3. Toxicological Analyses

Blood CO-Hb saturation (%) was analyzed using a CO-oximeter system (ABL80 FLEX system; Radiometer Corp., Tokyo, Japan). Blood cyanide and alcohol levels were determined using headspace gas chromatography/mass spectrometry [30,31]. Drug analyses were performed using gas chromatography/mass spectrometry [32].

### 2.4. Biochemical Analysis

Levels of PRL in serum and CSF were measured by chemiluminescent enzyme immunoassay using PATHFAST^®^ (LSI Medience, Tokyo, Japan) according to the manufacturer’s protocol [33]. The PRL concentrations in serum and CSF that were measured and reported in a previous study [21] were included in the present analysis.

### 2.5. Western Blotting

For Western blotting analysis, we used tissue from the parietal cerebral cortex, where many neurons are densely packed. The parietal lobe tissue specimens were immediately stored at −80 °C until use after collection for Western blotting analysis. Tissues were lysed in T-PER™ Tissue Protein Extraction Reagent (Thermo Fisher Scientific, Waltham, MA, USA) and Protease Inhibitor Cocktail Set V (FUJIFILM Wako Pure Chemical, Osaka, Japan), and the total amount of soluble protein was quantified using the Pierce™ BCA Protein Assay Kit (Thermo Fisher Scientific). Protein samples were separated by sodium dodecyl sulfate-polyacrylamide gel electrophoresis (Bolt™ 4–12% Bis-Tris Plus Gels; Thermo Fisher Scientific), and the resolved proteins were transferred onto nitrocellulose membranes (GE Healthcare, Buckinghamshire, UK). The membranes were blocked with Blocking One (Nacalai Tesque, Kyoto, Japan) and incubated with the primary antibodies followed by horseradish peroxidase-conjugated secondary antibody (Thermo Fisher Scientific). The primary antibodies were rabbit anti-human PRL polyclonal antibody (PB9361; Boster Biological Technology Co., Ltd., Pleasanton, CA, USA) and anti-β-actin mouse monoclonal antibody (AM4302; Thermo Fisher Scientific). The membranes were cut into two pieces; one piece was reacted with the primary antibody for PRL, and the other piece was reacted with the primary antibody for β-actin. After the staining steps, the two membrane pieces were photographed at the same time when immunoreactive signals were visualized by chemiluminescent detection using the ImageQuant LAS 500 (GE Healthcare). Protein quantitative analysis was performed with an ImageQuant TL (GE Healthcare); protein concentrations were normalized by calculating the ratios of PRL to β-actin.

### 2.6. Addition of PRL to Nerve Cells under Hypoxic Conditions

The amount of PRL in neuronal cells after the addition of PRL was compared under hypoxic and normoxic conditions in human neuroblastoma SH-SY5Y cells (EC94030304-F0; DS Pharma Biomedical, Suita, Japan). Cells were cultured in D-MEM/Ham’s F-12 medium supplemented with 1% MEM non-essential amino acids solution and 10% fetal bovine serum (Product No. 04-121-1A; Biological Industries Ltd., Kibbutz Beit-Haemek, Israel) and adjusted to 1.5 × 10^6^ cells per 35 mm culture dish. PRL treatment experiments were performed the next day. Cells were cultured in an incubator with 4.7% CO_2_ at 37 °C. PRL was added to the cell cultures at a final concentration of 20 ng/mL (*n* = 3 for each). After the addition of PRL, cells were cultured under hypoxic conditions with 1% O_2_ and 4.7% CO_2_ at 37 °C. At 30 min, 60 min, and 24 h after hypoxic exposure, cells were collected, and the PRL levels were analyzed by Western blotting.

### 2.7. Immunofluorescent Staining of Cultured Cells

SH-SY5Y cells (#EC94030304; KAC Co. Ltd., Kyoto, Japan) were cultured on chamber slides, then PRL (20 ng/mL) was added, and the cells were cultured for 30 min under hypoxic or normoxic conditions, followed by immunofluorescent staining. Cultured cells were washed with phosphate-buffered saline (PBS) and fixed with 4% paraformaldehyde phosphate buffer solution (FUJIFILM Wako Pure Chemical) for 10 min, then permeabilized with 0.5% Triton X-100 (PBS preparation) for 10 min at room temperature. A blocking solution (Blocking One Histo, Nacalai Tesque) was applied to cells at room temperature for 10 min. Next, mouse monoclonal anti-PRL antibody (SC-271773; Santa Cruz Biotechnology Inc., Santa Cruz, CA, USA) was added as the primary antibody, and the cells were incubated at 4 °C overnight. Subsequently, Cy5 goat anti-mouse IgG (A10524; Thermo Fisher Scientific), as the secondary antibody, reacted with the cells at room temperature for 60 min. The cells were sealed with a cover glass using a DAPI-containing encapsulation material (ProLong Gold Antifade Reagent with DAPI, #8961; Cell Signaling, Danvers, MA, USA) and evaluated using a fluorescence microscope (FSX100; Olympus, Tokyo, Japan).

### 2.8. Analysis of PRL Effects on Human Primary Neurons

Human primary neurons (HNs) prepared from human brains were obtained from ScienCell Research Laboratories (#1520; ScienCell, San Diego, CA, USA) and seeded onto 24-well cluster plates pretreated with Matrigel^®^ basement membrane matrix (Corning, Corning, NY, USA). Primary neurons were maintained in neuronal medium (ScienCell) supplemented with neuronal growth supplement (ScienCell) according to the manufacturer’s instructions [34]. To investigate the morphological changes in neurons induced by PRL, 1 to 100 ng/mL PRL was dropped into the medium, and the numbers of HN neurites were determined after 24 h of culture (*n* = 3 for each). As a control, medium with no added PRL was used. Neuronal maturation was evaluated using the ratio of cells of each neurite number (1 to 4 and ≥5) to all cells. Subsequently, to investigate the neurophysiological effects of PRL under hypoxia, 10 ng/mL PRL and 10 ng/mL T3 were dropped into the medium, respectively, and cultured under a hypoxic condition (3% O_2_). The percentage of dead cells due to apoptosis under hypoxia from 10 min to 24 h was measured by TUNEL staining (*n* = 3 for each). The ratio of nerve cell death was defined as the number of dead nerve cells/number of all nerve cells. As a control, medium with no added PRL or T3 was used.

### 2.9. Statistical Analysis

The Shapiro–Wilk and Kolmogorov–Smirnov tests were used to analyze the data distribution. Both tests similarly demonstrated that our dataset was not normally distributed. Spearman’s rank correlation coefficient was used for the comparisons of two values, including the PRL levels and the amount of brain PRL. For comparisons between groups, the nonparametric Mann–Whitney U test was used. In this test, the line in each box represents the median, and the lines outside each box represent the 90% confidence interval. The results of the cell culture experiments are presented as bar graphs, in which the bar indicates the mean, and the whiskers indicate the standard error. All analyses were performed using the SPSS 9.0 statistical package (SPSS Inc., Chicago, IL, USA). A *p*-value < 0.05 was considered significant.

## 3. Results

### 3.1. Amount of PRL in the Brain

**Figure 1:**
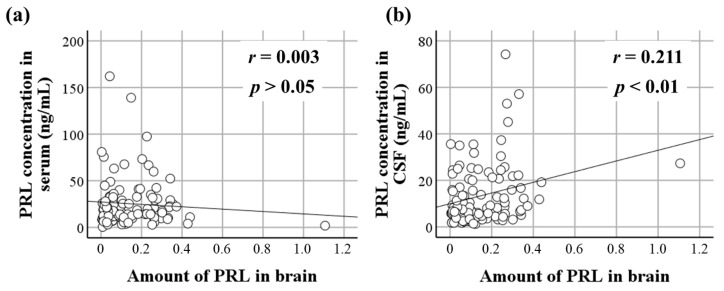
Relationships between the amount of PRL in the brain and (**a**) serum and (**b**) CSF levels of PRL. A slight correlation was observed between the CSF PRL levels and the amount of brain PRL (*r* = 0.211, *p* < 0.01). No relationship was found between the serum PRL levels and the amount of brain PRL.

**Figure 2:**
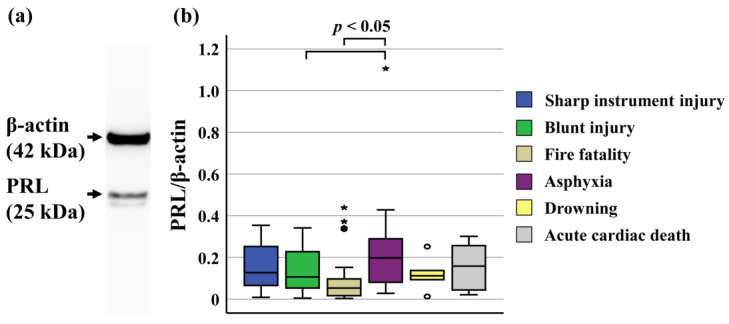
Analysis of the amount of PRL in the brain by Western blotting. (**a**) PRL protein was detected by Western blotting as a 25 kDa band. (**b**) Amount of PRL in the brain according to the cause of death. The amount of brain PRL was higher in cases of asphyxia than in cases of organ damage due to blunt injury and fire fatality (*p* < 0.05). The data are presented as box and whisker plots in which the central horizontal line in each box represents the median, the boxes span the interquartile range, and the whiskers represent the 90% confidence interval. Circles: outliers, asterisks: abnormal values.

There was no difference in the amount of brain PRL between the males and females. No relationship was found between the amount of brain PRL and the age, postmortem period, or survival period. Moreover, there were no differences between any of the survival-period groups (acute, subacute, and prolonged) among the sharp instrument injury and blunt injury cases. A slight correlation was observed between the CSF PRL levels and the amount of brain PRL (*r* = 0.211, *p* < 0.01). No relationship was found between the serum PRL levels and the amount of brain PRL (Figure 1). The amount of brain PRL was higher in cases of asphyxia than in cases of blunt injury and fire fatality (both *p* < 0.05). A trend was observed for PRL to be increased with asphyxia (Figure 2).

### 3.2. PRL Levels in SH-SY5Y Cells after Exposure to Hypoxic Conditions

**Figure 3:**
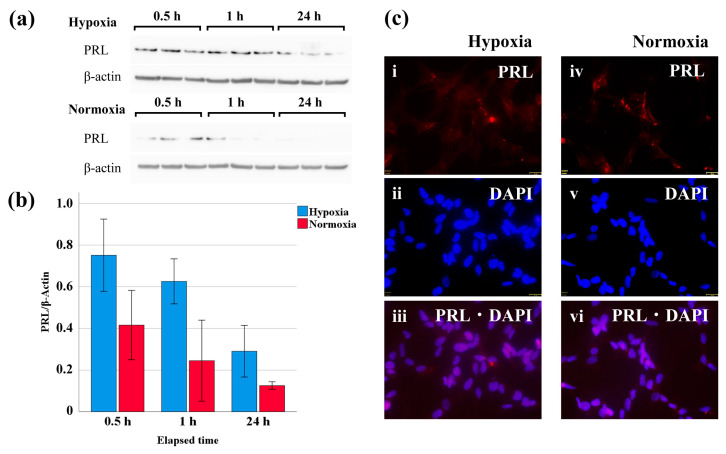
Western blot and immunofluorescent staining results of PRL-treated SH-SY5Y cells after exposure to hypoxia. (**a**) Western blotting for PRL and β-actin in SH-SY5Y cells. (**b**) PRL levels in PRL-treated SH-SY5Y cells after exposure to hypoxia. The data are presented as bar graphs in which the bars indicate the mean, and the whiskers indicate the standard error. (**c**) Photomicrographs showing the immunofluorescent staining of PRL in SH-SY5Y cells under hypoxic (**i**–**iii**) and normoxic (**iv**–**vi**) conditions.

After PRL was added to SH-SY5Y cells, a higher level of PRL was found in the cells exposed to hypoxic conditions than in those exposed to normoxic conditions. In addition, the concentration of PRL decreased over time for both the hypoxic and normoxic conditions (Figure 3a,b). On the other hand, immunofluorescent staining of PRL showed no significant difference in the fluorescence intensity between the hypoxic and normoxic conditions (Figure 3c).

### 3.3. Effects of PRL on Neuronal Maturation in Human Primary Neurons

**Figure 4:**
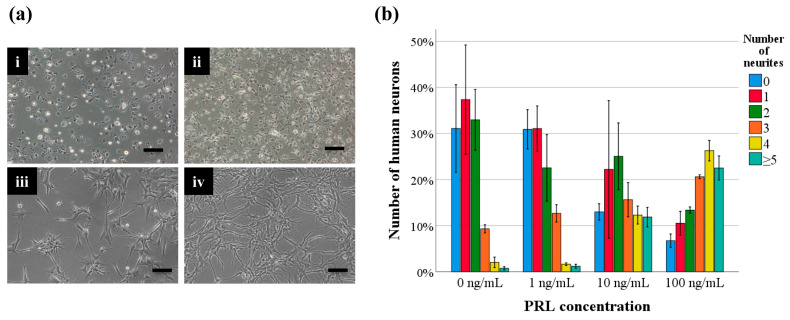
Effect of PRL concentration on the number of neurites. (**a**) HNs under phase-contrast microscopy. HNs were morphologically different in the medium with PRL [(**i**) 0 ng/mL, (**ii**) 1 ng/mL, (**iii**) 10 ng/mL, (**iv**) 100 ng/mL] after 24 h of culture. Bar = 100 µm. (**b**) Percentage of HNs of each neurite number after the addition of PRL. The data are presented as bar graphs in which the bars indicate the mean, and the whiskers indicate the standard error.

After 24 h of culture, morphological changes were observed in the HNs to which PRL was added. HNs without PRL did not have high neurite numbers and were immature, but as the concentration of added PRL increased, the number of neurites increased, and more morphologically mature neural cells were observed (Figure 4a).

The proportion of HNs with each neurite number after 24 h of culture was determined. Without the addition of PRL, the mean proportions were 0 neurites, 31.1%; 1 neurite, 37.3%; 2 neurites, 33.0%; 3 neurites, 9.3%; 4 neurites, 2.0%; and ≥5 neurites, 0.7%. With the addition of 1 ng/mL PRL, the mean proportions were 0 neurites, 30.9%; 1 neurite, 31.1%; 2 neurites, 22.5%; 3 neurites, 12.7%; 4 neurites, 1.6%; and ≥5 neurites, 1.2%. With the addition of 10 ng/mL PRL, the mean proportions were 0 neurites, 13.0%; 1 neurite, 22.2%; 2 neurites, 25.0%; 3 neurites, 15.6%; 4 neurites, 12.3%; and ≥5 neurites, 11.9%. With the addition of 100 ng/mL PRL, the mean proportions were 0 neurites, 6.7%; 1 neurite, 10.5%; 2 neurites, 13.4%; 3 neurites, 20.6%; 4 neurites, 26.3%; and ≥5 neurites, 22.5%. Of note, in HNs with the addition of PRL, many neurons with 0 to 3 neurites were observed, but in HNs to which 100 ng/mL of PRL was added, many neurons with 3 to 5 or more neurites were observed (Figure 4b).

### 3.4. Neuronal Protection of Human Primary Neurons under Hypoxic Conditions

The proportion of dead HNs due to apoptosis was determined for each time point. Without the addition of PRL, the mean proportion at each time point was 10 min, 5.3%; 30 min, 24.8%; 60 min, 53.0%; 3 h, 62.2%; 6 h, 83.1%; 12 h, 90.3%; and 24 h, 95.8%. With the addition of T3, the mean proportion at each time point was 10 min, 4.4%; 30 min, 20.9%; 60 min, 46.3%; 3 h, 50.4%; 6 h, 63.0%; 12 h, 75.7%; and 24 h, 80.3%. With the addition of PRL, the mean proportion at each time point was 10 min, 2.6%; 30 min, 15.9%; 60 min, 31.3%; 3 h, 38.6%; 6 h, 49.7%; 12 h, 61.3%; and 24 h, 71.7%.

**Figure 5:**
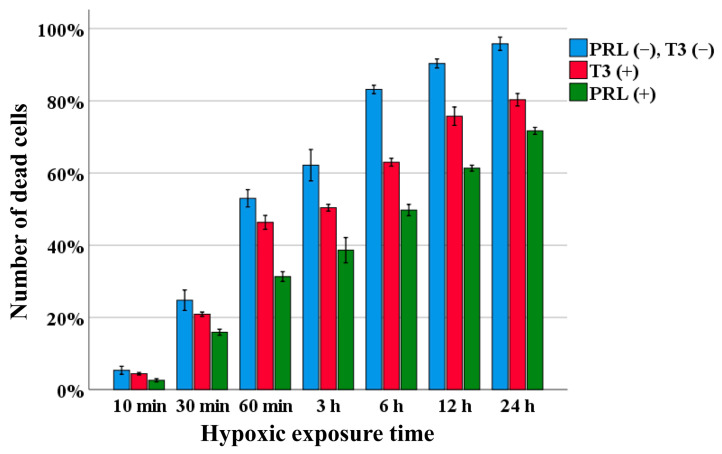
Neuroprotective functions of PRL and T3 in HNs under hypoxic conditions. The percentage of dead cells due to apoptosis from hypoxic exposure for 10 min to 24 h was measured by TUNEL staining. The data are presented as bar graphs in which the bars indicate the mean, and the whiskers indicate the standard error.

When the HNs were exposed to hypoxic conditions, cell death due to apoptosis was observed, and the number of apoptotic cells increased with longer hypoxic exposure time. Among the HNs without the addition of PRL, the percentage of dead cells was 90% or more after 24 h of hypoxic exposure. However, among the HNs with the addition of T3 or PRL, the percentage of dead cells was about 80% and 70%, respectively, after 24 h of hypoxic exposure. There were fewer apoptotic cells among the HNs with the addition of PRL or T3 than among the HNs without the addition of PRL or T3. Furthermore, PRL more strongly suppressed apoptosis due to hypoxia exposure than T3 (Figure 5).

## 4. Discussion

To determine whether PRL is present in the brain, we examined PRL in the brain of forensic autopsy cases. Western blot analysis using brain tissue samples showed that the amount of brain PRL correlated with CSF PRL. The amount of brain PRL was not influenced by sex, age, postmortem period, or survival period. Furthermore, the amount of PRL in the brain tended to increase with hypoxia, and PRL accumulated in the brain. Previous studies have reported that PRL is transported from the blood to the CSF in acute hypoxic/ischemic conditions [7,21]. It was suggested that PRL transported to the CSF may be taken into the brain as a result of acute systemic hypoxia due to asphyxia. On the other hand, experiments using SH-SY5Y cells showed that PRL was more abundant in cells exposed to hypoxia than those under normoxia. These results suggested that PRL accumulates in the neurons of the brain and is involved in the response to hypoxic conditions. The reason why no observable difference was seen in the immunofluorescence staining results between the hypoxic and normoxic conditions may be because of the small amount of PRL accumulated on a cell-by-cell basis or because PRL is disseminated within and around the cells.

The source of PRL in the brain has been studied extensively, but it remains controversial. Although sources of PRL other than the pituitary gland have been reported [8,9,10,11], PRL expression is very low in brain regions other than the pituitary gland, and it is unlikely that PRL produced in extrapituitary tissues has any effect on the circulating levels of PRL [13]. Therefore, the increase in PRL observed in this study during asphyxiation is believed to be due to pituitary-derived PRL crossing the blood–brain barrier and accumulating in neurons under specific physiological conditions (acute hypoxia). In this study, although PRL accumulation in the brain tended to be higher among the cases of asphyxia, no significant differences were observed for some of the other causes of death. This might be because of secondary hypoxia due to blood circulation failure [35]. Reportedly, the final stage of drowning involves alveolar damage rather than respiratory failure [36]. However, by simply measuring the amount of PRL in the brain, acute cardiac death or drowning was difficult to consider as a control for deaths that include brain lesioning or decreased oxygen.

With the addition of PRL to HNs, we found that the number of neurites increased after 24 h of culture as the PRL concentration increased. This finding suggested that PRL promotes the growth of neuronal cells. Furthermore, in the HN hypoxic exposure experiment, the proportion of apoptotic cells among the HNs with the addition of PRL or T3 was lower than that among the HNs without the addition of PRL or T3. This suggested that T3 and PRL have neuroprotective activity against hypoxia. In addition, PRL more strongly suppressed hypoxia-induced apoptosis in the HNs than T3, suggesting that PRL has a stronger neuroprotective effect than T3.

Thyroid hormone is known to play an important role in neurodevelopment [37]. The neuroprotective effects of thyroid hormone treatment after acute brain injury, including stroke and traumatic brain injury, have been reported [38,39]. Lin et al. showed that T3 significantly reduced the production of reactive oxygen species and prevented neuronal cell death by promoting the removal of damaged mitochondria by Pink1-dependent mitophagy [37]. In the in vitro experiments of the present study, we also found a similar protective effect against neuronal cell death in a hypoxic environment. Regarding the neuroprotective effects of PRL, it has been reported that pretreatment of rat hippocampal neurons with PRL before glutamate incubation prevented cell death and mitochondrial dysfunction [40]. In addition, the JAK-STAT signaling pathway may be involved in a mechanism by which PRL suppresses neuronal apoptosis under hypoxic conditions [41]. PRL is known to activate the JAK-STAT signaling pathway through PRLR, and the JAK-STAT signaling pathway is involved in apoptosis [42]. It has been reported that PRL-induced apoptotic effects are mediated by the JAK2/STAT5 pathway [13]. It has also been reported that PRL may regulate anti-apoptotic gene induction via STAT3 activation in cervical cancer cells [43]. Thus, apoptosis may also be suppressed by a STAT-mediated mechanism in human neurons.

Our results showed that PRL protects neurons from hypoxic environments but that the neuroprotective mechanism appears to differ from that of T3. PRL showed a stronger neuroprotective effect than T3, indicating a difference in their neuroprotective mechanisms. Nonetheless, the neuroprotective effects of PRL are complex, and research remains limited [34]. Elucidation of the neuroprotective properties of PRL may facilitate its application as a neuroprotective peptide for the treatment of HIE in the future.

This study has several limitations. In this study, asphyxia was used as a condition indicating acute systemic hypoxia. Asphyxia causes an acute hypoxic state and is known to have a particularly large effect on the brain [44]. However, research using autopsy tissues has limitations in terms of the sample size and analysis methods, and comparisons with research using cultured cells would be more appropriate for achieving the research objectives. Nonetheless, the cells used in this study included primary cultured human nerve cells, which are speculated to better reflect living nerve cells than established cell lines, such as other neuroblastoma cells. In addition, in this study, the amount of PRL present in the brain was measured, and the possibility that PRL accumulates in the brain under hypoxia was raised. However, some papers have reported that PRL is expressed in part of the brain [8,9,10,11], but it was not possible for us to determine whether the PRL found in the brain was derived from the brain itself or was transported from the blood. In addition, cell-to-cell relationships might differ in autopsy samples and monocultures, but we were unable to assess these relationships. To address these limitations, it may be necessary to conduct analyses of the PRL levels and PRLR in various regions of the brain. Such analyses may allow us to better understand the influence of potential confounders and further elucidate the role of PRL in hypoxia. In addition, the use of a co-culture system may be useful for culturing and analyzing not only nerve cells but also other types of cells within the central nervous system, such as microglia and astrocytes.

## 5. Conclusions

In conclusion, the present study results suggest that PRL accumulates in the brain during hypoxia, potentially influences neuronal activity, and exhibits neuroprotective properties against hypoxia-induced neuronal injury. However, the neuroprotective effects of PRL are complex, and research remains limited. Further elucidation of the mechanisms underlying the effects of PRL on neural responses to hypoxia may require a better understanding of the potential confounders and influences of cell-to-cell relationships. In addition, elucidation of the neuroprotective properties of PRL may facilitate its application as a neuroprotective peptide for the treatment of HIE.

## Figures and Tables

**Table 1:** Case profiles (*n* = 104).

Cause of Death		*n*	Male/Female	Age (Years)		Survival Period (h)	Postmortem Period (h)	
				Range	Median		Range	Median
Sharp instrument injury	Acute	5	1/4	46–86	62	<0.5	12–64	23
	Subacute	7	7/0	47–93	70	3–6	8–35	30
Blunt injury	Acute	7	7/0	20–84	63	<0.5	8–49	29
	Subacute	14	9/5	47–100	59	2–6	7–58	26
	Prolonged	11	8/3	1–93	71	7–24	15–73	29
Fire fatality	CO-Hb < 30%	6	5/1	44–86	71.5	<0.5	15–36	19
	CO-Hb = 30–60%	12	8/4	51–88	72	<0.5	11–40	22
	CO-Hb > 60%	8	6/2	61–79	74.5	<0.5	9–38	17
Asphyxia	Hanging	6	4/2	49–70	56.5	0.5–3	17–61	39
	Strangulation	4	2/2	37–87	68.5	<0.5	19–65	47
	Others	10	8/2	0–86	67.5	0.5–6	8–57	29
Drowning		6	6/0	33–85	64.5	<0.5	13–61	32
Acute cardiac death		8	8/0	0–75	45	<0.5	16–37	26
Total		104	82/22	0–100	67	<0.5–24	7–73	27

## Data Availability

The original contributions presented in this study are included in the article. Further inquiries can be directed to the corresponding author.

## References

[1] Bernard V., Young J., Binart N. (2019). Prolactin-a pleiotropic factor in health and disease. Nat. Rev. Endocrinol..

[2] Jara L.J., Medina G., Saavedra M.A., Vera-Lastra O., Torres-Aguilar H., Navarro C., Vazquez Del Mercado M., Espinoza L.R. (2017). Prolactin has a pathogenic role in systemic lupus erythematosus. Immunol. Res..

[3] Shingo T., Gregg C., Enwere E., Fujikawa H., Hassam R., Geary C., Cross J.C., Weiss S. (2013). Pregnancy-stimulated neurogenesis in the adult female forebrain mediated by prolactin. Science.

[4] Vanoye-Carlo A., Morales T., Ramos E., Mendoza-Rodríguez A., Cerbón M. (2008). Neuroprotective effects of lactation against kainic acid treatment in the dorsal hippocampus of the rat. Horm. Behav..

[5] Larsen C.M., Grattan D.R. (2012). Prolactin, neurogenesis, and maternal behaviors. Brain Behav. Immun..

[6] Cabrera-Reyes E.A., Limón-Morales O., Rivero-Segura N.A., Camacho-Arroyo I., Cerbón M. (2017). Prolactin function and putative expression in the brain. Endocrine.

[7] Walsh R.J., Slaby F.J., Posner B.I. (1987). A receptor-mediated mechanism for the transport of prolactin from blood to cerebrospinal fluid. Endocrinology.

[8] Nogami H., Hoshino R., Ogasawara K., Miyamoto S., Hisano S. (2007). Region-specific expression and hormonal regulation of the first exon variants of rat prolactin receptor mRNA in rat brain and anterior pituitary gland. J. Neuroendocrinol..

[9] Emanuele N.V., Jurgens J.K., Halloran M.M., Tentler J.J., Lawrence A.M., Kelley M.R. (1992). The rat prolactin gene is expressed in brain tissue: Detection of normal and alternatively spliced prolactin messenger RNA. Mol. Endocrinol..

[10] Torner L., Toschi N., Nava G., Clapp C., Neumann I.D. (2002). Increased hypothalamic expression of prolactin in lactation: Involvement in behavioural and neuroendocrine stress responses. Eur. J. Neurosci..

[11] Roselli C.E., Bocklandt S., Stadelman H.L., Wadsworth T., Vilain E., Stormshak F. (2008). Prolactin expression in the sheep brain. Neuroendocrinology.

[12] Bugge K., Papaleo E., Haxholm G.W., Hopper J.T., Robinson C.V., Olsen J.G., Lindorff-Larsen K., Kragelund B.B. (2016). A combined computational and structural model of the full-length human prolactin receptor. Nat. Commun..

[13] Costa-Brito A.R., Gonçalves I., Santos C.R.A. (2022). The brain as a source and a target of prolactin in mammals. Neural Regen. Res..

[14] Neradugomma N.K., Subramaniam D., Tawfik O.W., Goffin V., Kumar T.R., Jensen R.A., Anant S. (2014). Prolactin signaling enhances colon cancer stemness by modulating Notch signaling in a Jak2-STAT3/ERK manner. Carcinogenesis.

[15] Mortlock R.D., Georgia S.K., Finley S.D. (2020). Dynamic Regulation of JAK-STAT Signaling Through the Prolactin Receptor Predicted by Computational Modeling. Cell Mol. Bioeng..

[16] Hathaway C.A., Rice M.S., Collins L.C., Chen D., Frank D.A., Walker S., Clevenger C.V., Tamimi R.M., Tworoger S.S., Hankinson S.E. (2023). Prolactin levels and breast cancer risk by tumor expression of prolactin-related markers. Breast Cancer Res..

[17] Knudtzon J., Bogsnes A., Norman N. (1989). Changes in prolactin and growth hormone levels during hypoxia and exercise. Horm. Metab. Res..

[18] Zhang Y.S., Du J.Z. (2000). The response of growth hormone and prolactin of rats to hypoxia. Neurosci. Lett..

[19] Richalet J.P., Letournel M., Souberbielle J.C. (2010). Effects of high-altitude hypoxia on the hormonal response to hypothalamic factors. Am. J. Physiol. Regul. Integr. Comp. Physiol..

[20] Patil M.J., Henry M.A., Akopian A.N. (2014). Prolactin receptor in regulation of neuronal excitability and channels. Channels.

[21] Tani N., Ikeda T., Watanabe M., Toyomura J., Ohyama A., Ishikawa T. (2018). Prolactin selectively transported to cerebrospinal fluid from blood under hypoxic/ischemic conditions. PLoS ONE.

[22] Allen K.A., Brandon D.H. (2011). Hypoxic ischemic encephalopathy: Pathophysiology and experimental treatments. Newborn Infant. Nurs. Rev..

[23] Johnston M.V., Fatemi A., Wilson M.A., Northington F. (2011). Treatment advances in neonatal neuroprotection and neurointensive care. Lancet Neurol..

[24] Barranco R., Bonsignore A., Ventura F. (2021). Immunohistochemistry in postmortem diagnosis of acute cerebral hypoxia and ischemia: A systematic review. Medicine.

[25] Oehmichen M., Meissner C., von Wurmb-Schwark N., Schwark T. (2003). Methodical approach to brain hypoxia/ischemia as a fundamental problem in forensic neuropathology. Leg. Med..

[26] Edwards A.B., Anderton R.S., Knuckey N.W., Meloni B.P. (2018). Perinatal hypoxic-ischemic encephalopathy and neuroprotective peptide therapies: A case for cationic arginine-rich peptides (CARPs). Brain Sci..

[27] Minato K., Tomimatsu T., Mimura K., Jugder O., Kakigano A., Kanayama T., Fujita S., Taniguchi Y., Kanagawa T., Endo M. (2013). Hypoxic preconditioning increases triiodothyronine (T3) level in the developing rat brain. Brain Res..

[28] Pathipati P., Gorba T., Scheepens A., Goffin V., Sun Y., Fraser M. (2011). Growth hormone and prolactin regulate human neural stem cell regenerative activity. Neuroscience.

[29] Paul D.A., Rodrigue A., Contento N., Haber S., Hoang R., Rahmani R., Hirad A., Shafiq I., Williams Z., Vates G.E. (2022). Prolactin at moderately increased levels confers a neuroprotective effect in non-secreting pituitary macroadenomas. PLoS ONE.

[30] Maeda H., Fukita K., Oritani S., Nagai K., Zhu B.L. (1996). Evaluation of post-mortem oxymetry in fire victims. Forensic Sci. Int..

[31] Maeda H., Zhu B.L., Ishikawa T., Oritani S., Michiue T., Li D.R., Zhao D., Ogawa M. (2006). Evaluation of post-mortem ethanol concentrations in pericardial fluid and bone marrow aspirate. Forensic Sci. Int..

[32] Tominaga M., Michiue T., Ishikawa T., Inamori-Kawamoto O., Oritani S., Maeda H. (2015). Evaluation of postmortem drug concentrations in cerebrospinal fluid compared with blood and pericardial fluid. Forensic Sci. Int..

[33] Sugie Y., Igami K., Shoji K., Arai N., Tazaki Y., Kouta H., Okamura Y., Tashiro S., Yokoi H. (2011). Performance evaluation of the new rapid fertility assays in whole blood and plasma on PATHFAST. Clin. Lab..

[34] Watanabe S., Ohno S., Shirogane Y., Suzuki S.O., Koga R., Yanagi Y. (2015). Measles virus mutants possessing the fusion protein with enhanced fusion activity spread effectively in neuronal cells, but not in other cells, without causing strong cytopathology. J. Virol..

[35] Rahaman P., Del Bigio M.R. (2018). Histology of Brain Trauma and Hypoxia-Ischemia. Acad. Forensic Pathol..

[36] Miyazato T., Ishikawa T., Michiue T., Maeda H. (2012). Molecular pathology of pulmonary surfactants and cytokines in drowning compared with other asphyxiation and fatal hypothermia. Int. J. Leg. Med..

[37] Lin C., Li N., Chang H., Shen Y., Li Z., Wei W., Chen H., Lu H., Ji J., Liu N. (2020). Dual effects of thyroid hormone on neurons and neurogenesis in traumatic brain injury. Cell Death Dis..

[38] Rastogi L., Godbole M.M., Sinha R.A., Pradhan S. (2018). Reverse triiodothyronine (rT3) attenuates ischemia-reperfusion injury. Biochem. Biophys. Res. Commun..

[39] Liu Y.Y., Brent G.A. (2018). Thyroid hormone and the brain: Mechanisms of action in development and role in protection and promotion of recovery after brain injury. Pharmacol. Ther..

[40] Rivero-Segura N.A., Flores-Soto E., García de la Cadena S., Coronado-Mares I., Gomez-Verjan J.C., Ferreira D.G., Cabrera-Reyes E.A., Lopes L.V., Massieu L., Cerbón M. (2017). Prolactin-induced neuroprotection against glutamate excitotoxicity is mediated by the reduction of [Ca^2+^]i overload and NF-κB activation. PLoS ONE.

[41] Radhakrishnan A., Raju R., Tuladhar N., Subbannayya T., Thomas J.K., Goel R., Telikicherla D., Palapetta S.M., Rahiman B.A., Venkatesh D.D. (2012). A pathway map of prolactin signaling. J. Cell Commun. Signal.

[42] Nicolas C.S., Amici M., Bortolotto Z.A., Doherty A., Csaba Z., Fafouri A., Dournaud P., Gressens P., Collingridge G.L., Peineau S. (2013). The role of JAK-STAT signaling within the CNS. JAKSTAT.

[43] Ramírez de Arellano A., Lopez-Pulido E.I., Martínez-Neri P.A., Estrada Chávez C., González Lucano R., Fafutis-Morris M., Aguilar-Lemarroy A., Muñoz-Valle J.F., Pereira-Suárez A.L. (2015). STAT3 activation is required for the antiapoptotic effects of prolactin in cervical cancer cells. Cancer Cell Int..

[44] Zeng Y., Lv Y., Tao L., Ma J., Zhang H., Xu H., Xiao B., Shi Q., Ma K., Chen L. (2016). G6PC3, ALDOA and CS induction accompanies mir-122 down-regulation in the mechanical asphyxia and can serve as hypoxia biomarkers. Oncotarget.

